# Telemedicine in pediatric rheumatology: the video pediatric gait, arms, legs, and spine (v-pGALS) examination

**DOI:** 10.55730/1300-0144.5874

**Published:** 2024-07-02

**Authors:** Zeynep BALIK, Seher ŞENER, Yağmur BAYINDIR, Müşerref KASAP CÜCEOĞLU, Emil ALİYEV, Özge BAŞARAN, Yelda BİLGİNER, Seza ÖZEN, Ezgi Deniz BATU

**Affiliations:** Department of Pediatric Rheumatology, Faculty of Medicine, Hacettepe University, Ankara, Turkiye

**Keywords:** Pediatric rheumatology, pGALS, telemedicine, video pGALS

## Abstract

**Background/aim:**

Video pediatric gait, arms, leg, and spine (v-pGALS) is a virtual application of the pediatric gait, arms, leg, and spine (pGALS) examination performed by video. We aimed to verify the applicability, validity, and accuracy of the Turkish translation of v-pGALS in a large pediatric patient cohort.

**Materials and methods:**

Children aged 4–18 years seen between May and June 2022 were included. A hands-on physical examination and v-pGALS were performed. Demographics, active symptoms, physical examination findings, diagnosis, and v-pGALS findings were recorded. The acceptability of v-pGALS, in terms of additional distress and duration, was measured by the parent/patient using a visual analog scale (VAS).

**Results:**

102 patients (median age 12.41 years) were included. Juvenile idiopathic arthritis (JIA) was the most common diagnosis. The median duration of v-pGALS was 7 min. An abnormal v-pGALS was identified in 25 patients while the hands-on physical examination was abnormal in 27 patients. Scoliosis and pes planus were missed in v-pGALS. Both children and parents gave a median VAS score of 0 for additional discomfort and duration. That is, the duration of v-pGALS was acceptable for ≥98% of the patients/parents, and ≥98% mentioned that it caused little/no discomfort. The sensitivity and specificity of v-pGALS were 92.6% and 100%, respectively, for the detection of musculoskeletal (MSK) abnormalities.

**Conclusion:**

The v-pGALS is an applicable, accurate, and practical tool for evaluating MSK problems in children. The Turkish translation was also conveniently acceptable.

## 1. Introduction

The severe acute respiratory syndrome coronavirus 2 (SARS-CoV-2) pandemic caused rapid and significant changes in medical care all over the world. The precautions introduced by the pandemic, such as quarantine, social distancing measures, and the requirement of proving COVID-19-free status to cross district borders, limited patient access to healthcare [1,2]. In this situation, telemedicine stood out as a method to secure the continuity of health care [3].

Before the pandemic, telemedicine was not frequently used in pediatric rheumatology practice [4]. It was only used when the patient was living in a remote area and could not reach face-to-face medical care [5]. In most children with rheumatic diseases, both disease and treatment may lead to immune dysregulation, which raises concerns about severe infection or infection complications among pediatric rheumatology patients and their parents which leads to delays in clinical appointments [1,6]. Thus, telemedicine was quickly incorporated into the routine practice of pediatric rheumatology during the pandemic [7].

Pediatric gait, arms, leg and spine (pGALS) is a practical and simple method used for evaluating the musculoskeletal (MSK) system in children [8]. A medical student, primary care physician, or general pediatrician can easily evaluate MSK abnormalities using pGALS. It has been validated for children aged 4–16 years, and it is highly sensitive and specific in detecting MSK abnormalities [9]. It has been translated into many languages, including Turkish, and has been shown to be acceptable and practical [10,11]. Video pediatric gait, arms, leg, and spine (v-pGALS) is the virtual version of pGALS. It was first developed by Shenoi et al. [7] to evaluate musculoskeletal functioning during telemedicine visits for pediatric rheumatology patients in 2020.

V-pGALS has some differences from the original pGALS examination, the most significant being that v-pGALS is performed by the patient and the parents according to the doctor’s instructions, while pGALS is performed by the physician. During v-pGALS, passive joint movements, knee crepitation, and joint tenderness examinations are performed by the parents. In v-pGALS, screening questions include morning stiffness (in minutes) and a pain scale of 0–10 in addition to the pGALS screening questions. Furthermore, the v-PGALS includes some examinations that are not involved in pGALS. Firstly, the gait examination includes a balance test on tiptoes. The arms examination includes a movement called a ‘cat claw’ or ‘monkey paw’ for observing the flexion of the small joints of fingers. Additionally, the metacarpophalangeal joint tenderness is graded on a scale from 0 to 10. The legs examination includes standing on one leg, doing squats and sitting cross-legged, which aid understanding of fluency, balance, proximal muscle strength, and active hip, knee, and ankle movement. Leg rolls while supine and ‘W’ and ‘X’ leg positions while prone check internal and external hip rotation. Moving the jaw side-to-side checks temporomandibular joint movements. The final examination, not included in pGALS, is a measurement of sacroiliac joint tenderness, which can be performed while the child is lying prone or standing.

V-pGALS is used for evaluating the MSK system during telemedicine visits [7]. It facilitates access to healthcare and reduces travel time and health costs [12]. It also reduces the SARS-CoV-2 contact risk of face-to-face care [13].

A pilot study performed at a physical medicine and rehabilitation unit tested the practicality and acceptability of the Turkish translation of v-pGALS. However, the number of patients was limited (n = 20), the accuracy of v-pGALS was not evaluated, and the setting was a physical medicine and rehabilitation unit [14]. In this study, we aimed to do rheumatological assessment via telemedicine by performing v-pGALS and to evaluate the applicability, validity, and accuracy of the Turkish translation of the v-pGALS with a large patient cohort in the pediatric rheumatology department.

## 2. Materials and methods

### 2.1. Study design

Children aged 4–18 years who were followed up in the Hacettepe University Pediatric Rheumatology Clinic between May and June 2022 were included. Because p-GALS has been validated for children above four years of age [9], children below four years of age were excluded from the study. Additionally, patients above the age of 18 and those who did not agree to participate in the study were excluded. The demographics, active signs and symptoms, physical examination, diagnosis, and v-pGALS findings were noted for all participants. The translation–back-translation process was used to create the Turkish version of the v-pGALS [15]. A pediatric rheumatologist fluent in both English and Turkish translated the v-pGALS into Turkish, and another pediatric rheumatologist then retranslated it into English. An expert found the most suitable translation for each of the components and created a unified Turkish text.

The original v-pGALS is available at http://www.pmmonline.org/doctor/approach-to-clinical-assessment/examination/v-pgals. The Turkish translation of the v-pGALS is shown in the Supplementary Figure.

### 2.2. Performing v-pGALS

The patients participating in the study were first asked about muscle, back, or joint pain and morning stiffness. They were also asked about difficulties dressing and going up and down stairs. A pediatric rheumatologist performed the v-pGALS using a Zoom video interface connecting the doctor to the patients and their parents in a different room. The v-pGALS consists of 23 sections that assess pain, limitations in joint movement, joint disease in the lower and upper limbs, spine, and posture. Parents were asked to participate in the examination of joint range of motion and palpation. If at least one response is positive, the v-pGALS is considered to be positive. After v-pGALS, the physician performed a normal hands-on physical examination, which is the gold standard for positive findings. The v-pGALS was performed before the hands-on physical examination, and the physician performing the hands-on physical examination did not know the v-pGALS result. The acceptability of v-pGALS, in terms of additional distress and duration, was measured using a visual analog scale (VAS) with smiley faces. The pain was rated on a scale of 0 to 10, with 10 being the worst.

### 2.3. Ethics approval

The Hacettepe University Ethics Committee approved the study (approval number: GO 22/331; approval date, 10 May 2022), and all participants signed an informed consent form before participation.

### 2.4. Statistical analysis

The data were statistically analyzed using SPSS Statistics 26 (IBM Corp.; Armonk, NY, USA). Descriptive statistics were presented as maximum (max), median, minimum (min), frequency, and percentage. The variables were investigated analytically (Kolmogorov–Smirnov and Shapiro–Wilk’s tests) and visually (probability plots and histograms) to determine whether they were normally distributed. The Mann–Whitney U test was used for comparing nonnormally distributed continuous variables. The specificity, sensitivity, and negative and positive predictive values of the v-pGALS were computed based on comparison with the findings of the face-to-face examination. A p value less than 0.05 is considered statistically significant. A post hoc power analysis was conducted using G*power version 3.1.9.6.

## 3. Results

### 3.1. Patient characteristics

A total of 102 patients participated in the study. Twenty-two (21.6%) patients had active complaints. Juvenile idiopathic arthritis (JIA) was the most common diagnosis (n = 26; 25.5%), followed by familial Mediterranean fever (FMF) (n = 19; 18.6%). The characteristics of the patients are presented in Table 1.

### 3.2. Screening questions of v-pGALS

Twenty-two (21.6%) patients answered positively to at least one of three v-pGALS screening questions (Table 2). Twenty-one (20.6%) patients had pain in the joints, muscles, or back. Three of those (2.9%) also had morning stiffness. The median (min–max) pain score was 4 (1–8). The median (min–max) morning stiffness duration was 20 (10–30) min. Two (2%) patients had difficulties going up or down the stairs. None of the patients had difficulty lifting an object or getting dressed. Of the 22 patients who answered positively to at least one screening question, the v-pGALS was abnormal for 12 (54.5%), whereas the hands-on physical examination was abnormal for 14 (63.6%). The sensitivity and specificity for the positive response to one or more v-pGALS screening questions to detect abnormal physical conditions were 51.8% and 89.3%, respectively. The question about pain had the highest sensitivity (48.1%) and the specificity was 89.3%. The sensitivity and specificity rates of questions about climbing stairs were 7.4% and 100%, respectively.

### 3.3 Performing v-pGALS

Abnormal v-pGALS was detected in 25 (24.5%) patients while the hands-on physical examination was abnormal in 27 (26.4%) patients. With v-pGALS, we were unable to detect abnormal physical examination findings in 2 (2%) patients. These patients had pes planus and scoliosis. All abnormal findings in the v-pGALS were also detected by the hands-on physical examination. The sensitivity and specificity of v-pGALS for detecting MSK abnormalities were 92.6% and 100%, respectively. The positive predictive value was 100%, while the negative predictive value was 97.4%.

The median (mix–max) duration of the v-pGALS examination was 7 (5–13) min, with positive v-pGALS lasting longer than negative v-pGALS (7 vs. 6 min; p < 0,001). A post hoc power analysis revealed a calculated power of 0.99, at a confidence level of 95%.

### 3.4. Evaluating v-pGALS

The pediatric patients and their parents gave a median (min–max) VAS score of 0 (0–5) and 0 (0–6) for additional discomfort and duration, respectively. That is, the duration of v-pGALS was acceptable for ≥98% of the patients and parents, and ≥98% mentioned that it led to little or no discomfort.

## 4. Discussion

This is the first and largest study in which the applicability, validity, and accuracy of the Turkish translation of the v-pGALS were evaluated by pediatric rheumatologists. The results demonstrated that the v-pGALS is an easy, quick, and acceptable tool for evaluating MSK problems in children with high sensitivity (92.6%) and specificity (100%). Also, the Turkish version of v-pGALS was acceptable in terms of duration and additional discomfort. The duration of the v-pGALS examination was acceptable for the majority of the patients and parents and they mentioned that it led to little or no discomfort. The main strength of this study is the high number of patients; the high statistical power due to the sufficient sample size increases the study’s significance.

There has been only one previous study on the Turkish translation of v-pGALS. Giray et al. [14], investigated the acceptability, practicality, and interrater reliability of the Turkish translation of real-time v-pGALS. They performed this study in a physical therapy and rehabilitation clinic with fewer patients (n = 20) and with other diseases, including rheumatic diseases, while the present study was performed in the pediatric rheumatology clinic with a large patient population and mostly in patients with a diagnosis of rheumatic disease. Giray et al. reported a median (mix–max) duration of v-pGALS as 15.8 (9–26) min, while it was shorter (median 7 min) in the present study. The duration might have varied based on the experience of the health professional performing v-pGALS. In our study, the majority (≥98%) of patients and parents found the duration acceptable and mentioned that v-pGALS caused little or no discomfort, whereas Giray et al. reported this as 95% [14]. In addition, they reported the sensitivity and specificity of v-pGALS as 93.75% and 100%, respectively, which were similar to our results (92.6% and 100%, respectively).

Video pGALS is the form of pGALS performed on video, with a few differences from the original pGALS. We previously reported a median (mix–max) duration of pGALS as 4 (2–12) min, which is shorter than the duration we found for v-pGALS [10]. The sensitivities and specificities of pGALS and v-pGALS are similar (93.7% vs. 92.6% and 97.4% vs. 100%, respectively). The longer duration of v-pGALS could be due to the challenges of guiding patients and parents via a video connection.

A Colombian study included 169 patients (mean age 9.43 years) to determine the usefulness of pGALS as a screening test in children [16]. It was found that a positive response to the first question in the examination had a sensitivity of 91.3% and a specificity of 53.3% for indicating MSK abnormalities in the examination. On the other hand, positive responses to any of these three questions had a sensitivity of 58%, a specificity of 94%, a positive likelihood ratio of 9.3, and a negative likelihood ratio of 0.44. The pGALS examination was found acceptable by 95.3% of patients. The mean duration of pGALS was 2.27 min. Compared to previous studies, the sensitivity and specificity of the positive response to one or more v-pGALS screening questions for detecting abnormal physical examinations were slightly lower in this study, at 51.8% and 89.3%, respectively.

In an emergency department in Peru, the Abernethy group [11] evaluated the Spanish translation of pGALS with 53 children aged 4 to 16 years. They found that the sensitivity and specificity of the positive response to one or more screening questions were 63.6% and 87.1% respectively. The higher sensitivity may be related to the fact that patients go to the emergency room when their pain worsens. In a study in Mexico, a total of 175 children aged 6 to 16 years (87 patients with osteomuscular disease and 88 healthy controls) underwent pGALS with a sensitivity of 97%, a specificity of 93%, and a positive odds ratio of 14.3% for the diagnosis of MSK disease [17].

There are some advantages to using v-pGALS. Most obviously, it provides patients living in remote areas access to needed healthcare [18]. It also reduces travel time and cost and lessens work and school absences [4,19]. It makes services like prescription renewals and appointment scheduling easier [20]. Aydemir et al. [21] provided health services through telemedicine for the follow-up of chronic childhood diseases in five different pediatric departments. They performed 263 interviews and evaluated these interviews according to the perspectives of patients, parents, and physicians. They showed that telemedicine reduced wasted time for transportation, days off from work and school, and waiting time to see a physician. Besides, they noted that telemedicine could be used for emergency visits as well as regular visits [21]. In another study from India, a group of researchers aimed to explore parental approaches to healthcare facilities to meet children’s surgical care needs during the COVID pandemic. In this study, they conducted an interview using a semi-structured interview program among 26 parents of children needing perioperative surgical care at a tertiary hospital. They found that telemedicine helped parents meet their children’s surgical care needs [2].

Despite these advantages, the v-pGALS has also some limitations. The most important problem is that subtle examination findings could be missed. In the present study, in two patients with abnormal physical examination (scoliosis and pes planus), these abnormalities were overlooked with v-pGALS. Because scoliosis and pes planus do not cause localized pain or swelling, they cannot be detected by palpation. The only way to detect these is by careful observation. Therefore, mild cases may be missed when using v-pGALS. It is also possible that the effectiveness of v-pGALS can be affected by the technological competence of both the clinicians and the patients/parents. Other limitations of the procedure are medical data security concerns, adequacy of the internet infrastructure, uncertainty about reimbursement conditions, and the challenges of long-term follow-up [22].

When using the v-pGALS procedure, there are some situations that the physician may encounter and should be careful about. Especially for patients whose history is given by the caregiver, complaints may be nonspecific and vague; the patients may have difficulty in localizing or describing pain in terms that adults understand [23]. This condition may affect the answers given to the questions in the v-pGALS. Therefore, the physician who uses v-pGALS in these situations may have difficulties. To minimize this possibility, the physician should be experienced with the child’s normal physiological and psychological development and the patterns of self-expression at different ages. Furthermore, proper interpretation of v-pGALS requires knowledge of normal ranges of joint motion in different age and ethnic groups, searching for asymmetry, and careful examination for subtle abnormalities [24]. It is also important to check for both verbal and nonverbal discomfort cues that may suggest joint pathology.

The most important limitation of our study was that different people performed the hands-on physical examinations and the v-pGALS. The v-pGALS was performed by a pediatric rheumatologist with two years of experience, while the hands-on physical examination was performed by an expert pediatric rheumatologist. For the patients whose abnormal findings could not be detected using v-pGALS, the abnormalities were more likely to have been detected if the examination had been performed by an expert. However, just like pGALS, v-pGALS is a tool designed for trainee use. Another study limitation was the low number of participants with MSK involvement, despite being a cohort study with a relatively large number of patients. This may affect the generalization of the study results.

In conclusion, the v-pGALS is an easy, quick, and acceptable tool for evaluating MSK problems in children. In terms of duration and additional discomfort, the Turkish version of v-pGALS was also acceptable. The results showed that v-pGALS might miss pathologies such as pes planus and scoliosis. Therefore, physicians should be more careful when evaluating these findings. Nevertheless, v-pGALS can detect most physical examination abnormalities, and it proved to be a useful tool for the uninterrupted follow-up of children with rheumatic diseases during the pandemic restrictions.

## Supplementary Information

Supplementary Figure Turkish translation of video pGALS

## Figures and Tables

**Table 1:** Characteristics of patients evaluated with v-pGALS.

Characteristics	Patients (n = 102), n (%)
Age, median (min–max), years	12.41 (4.23–17.84)
Sex, female	56 (54.9)
Active complaints	22 (21.6)
Ankle pain	10 (9.8)
Back pain	5 (4.9)
Leg pain	4 (3.9)
Hip pain	1 (1)
Weakness	1 (1)
Widespread joint pain	1 (1)
**Final diagnosis**	**n (%)**
JIA	26 (25.5)
FMF	19 (18.6)
FMF + JIA	6 (5.9)
FMF + PAN	1 (1)
FMF + IgAV	1 (1)
PFAPA	8 (7.8)
CNO	6 (5.9)
Growing pains	4 (3.9)
ANA positivity	4 (3.9)
SLE	3 (2.9)
Recurrent aphthous stomatitis	3 (2.9)
IgAV	3 (2.9)
IgAV + JIA	1 (1)
DADA2	2 (2)
JDM	2 (2)
Undifferentiated autoinflammatory disease	2 (2)
Scleroderma	2 (2)
MIS-C	2 (2)
PAN	1 (1)
Reynaud syndrome	1 (1)
Sjogren’s syndrome	1 (1)
Behcet’s disease	1 (1)
AAV	1 (1)
MCTD	1 (1)
Osgood–Schlatter disease	1 (1)

AAV: Antineutrophil cytoplasmic antibody-associated vasculitis; ANA: Antinuclear antibody; CNO: Chronic nonbacterial osteitis; DADA2: Deficiency of adenosine deaminase 2; FMF: Familial Mediterranean fever; IgAV: Immunoglobulin A vasculitis; JDM: Juvenile dermatomyositis; JIA: Juvenile idiopathic arthritis; MCTD: Mixed connective tissue disease; MIS-C: Multisystem inflammatory syndrome in children; PAN: Polyarteritis nodosa; PFAPA: Periodic fever, aphthous stomatitis, pharyngitis and cervical adenitis syndrome; SLE: Systemic lupus erythematosus

**Table 2:** Characteristics of the v-pGALS.

v-pGALS items	Patient results^a^ (n = 102), n (%)

Screening questions	
• Pain or stiffness in joints, muscles, or back?	11 (10.8)
• Difficulty getting dressed or lifting an object above shoulder level?	0
• Problem going up and down steps, squatting, or walking?	2 (2)

Gait	7 (6.9)
Appearance	6 (5.9)
Movement	3 (2.9)

Arms	9 (8.8)
Appearance	9 (8.8)
Movement	5 (4.9)

Legs	20 (19.6)
Appearance	17 (16.6)
Movement	12 (11.8)

Spine	1 (1)
Appearance	1 (1)
Movement	1 (1)

a Numbers and percentages represent positive responses to screening questions or abnormal examination findings.
