# Erratum to “ Telemedicine in pediatric rheumatology: the video pediatric gait, arms, legs, and spine (v-pGALS) examination” [Turkish Journal of Medical Sciences 54 (5) 2024 963–969 ]

**DOI:** 10.55730/1300-0144.5976

**Published:** 2025-03-01

**Authors:** Zeynep BALIK, Seher ŞENER, Yağmur BAYINDIR, Müşerref KASAP CÜCEOĞLU, Emil ALİYEV, Özge BAŞARAN, Yelda BİLGİNER, Seza ÖZEN, Ezgi Deniz BATU

**Affiliations:** Department of Pediatric Rheumatology, Faculty of Medicine, Hacettepe University, Ankara, Turkiye

This erratum is written to correct an error in our previous publication. Supplement Figure 2 was published incorrectly as one page instead of six.

Supplementary Figure Turkish translation of video pGALS

