# Supplementary figures and images for: Telemedicine in pediatric rheumatology: the video pediatric gait, arms, legs, and spine (v-pGALS) examination
Source: Turk J Med Sci. 2024 Jul 2;54(5):963–9. doi: 10.55730/1300-0144.5874 (PMC11518324; doi:10.55730/1300-0144.5874)

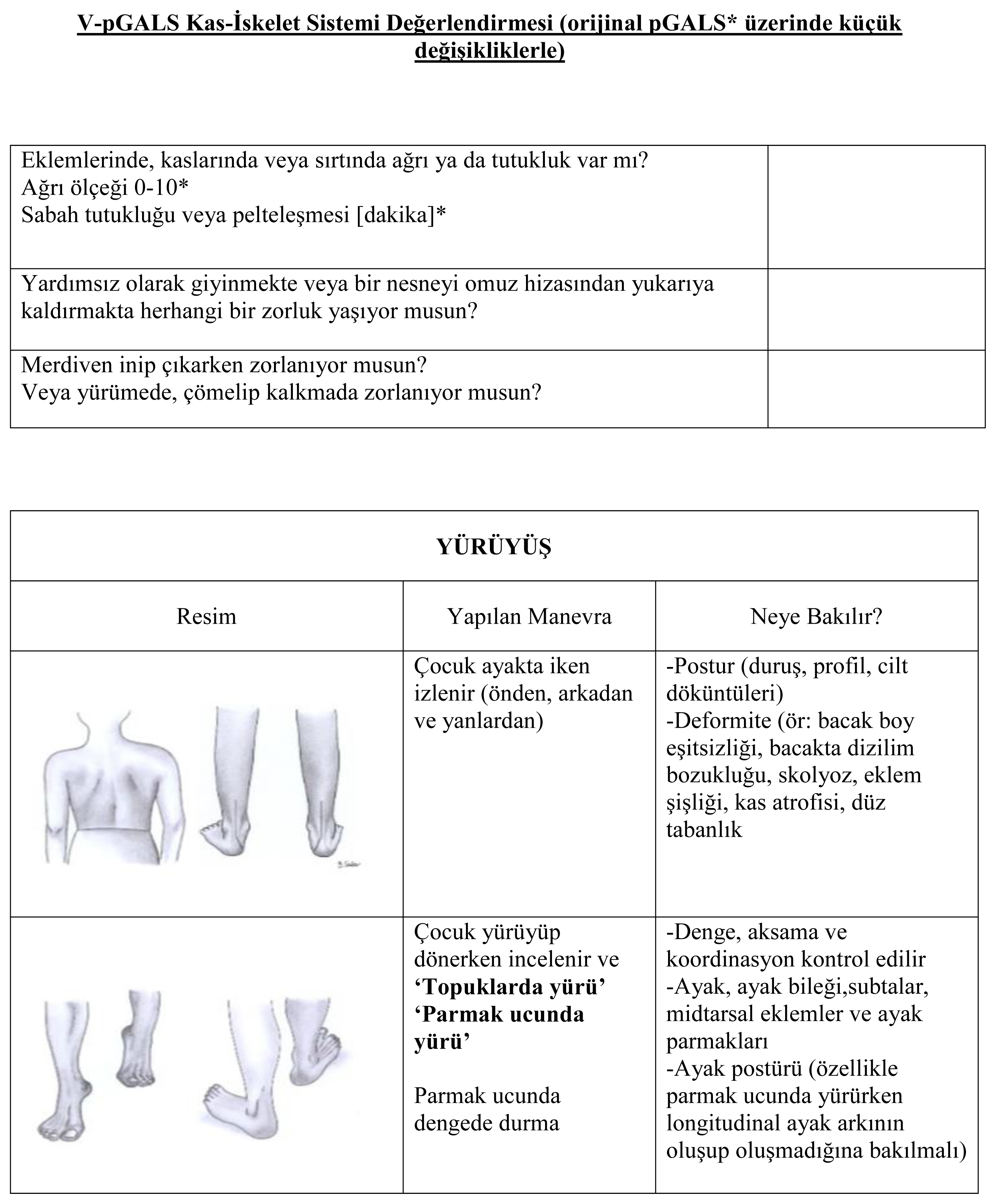

Supplement: Supplementary Figure — Turkish translation of video pGALS [file tjmed-54-05-963s1.tif]
