# Supplementary figures and images for: Erratum to “ Telemedicine in pediatric rheumatology: the video pediatric gait, arms, legs, and spine (v-pGALS) examination” [Turkish Journal of Medical Sciences 54 (5) 2024 963–969 ]
Source: Turk J Med Sci. 2025 Mar 1;55(1):348. doi: 10.55730/1300-0144.5976 (PMC11913511; doi:10.55730/1300-0144.5976)

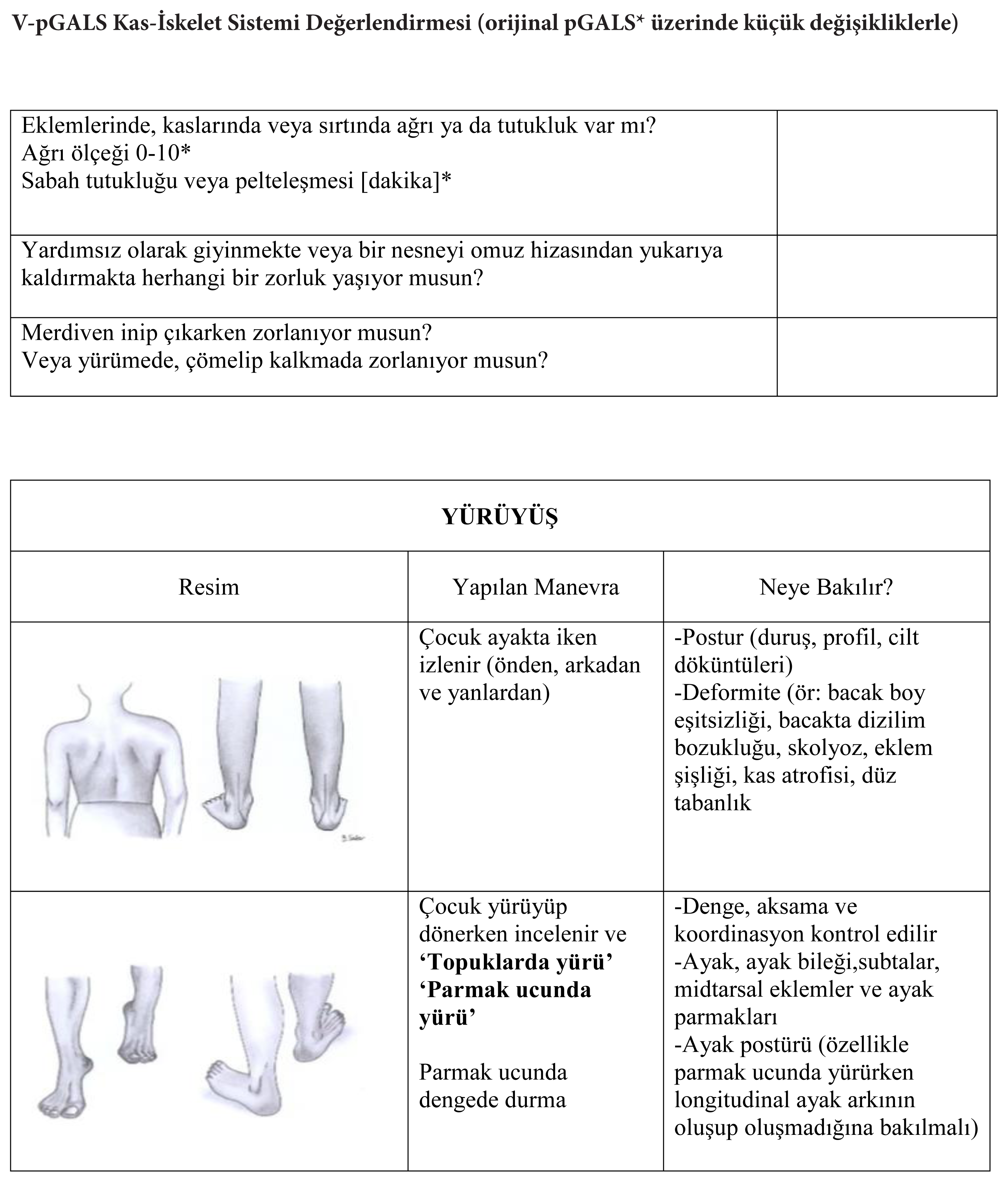

Supplement: Supplementary Figure — Turkish translation of video pGALS [file tjmed-55-01-348s1a.tif]

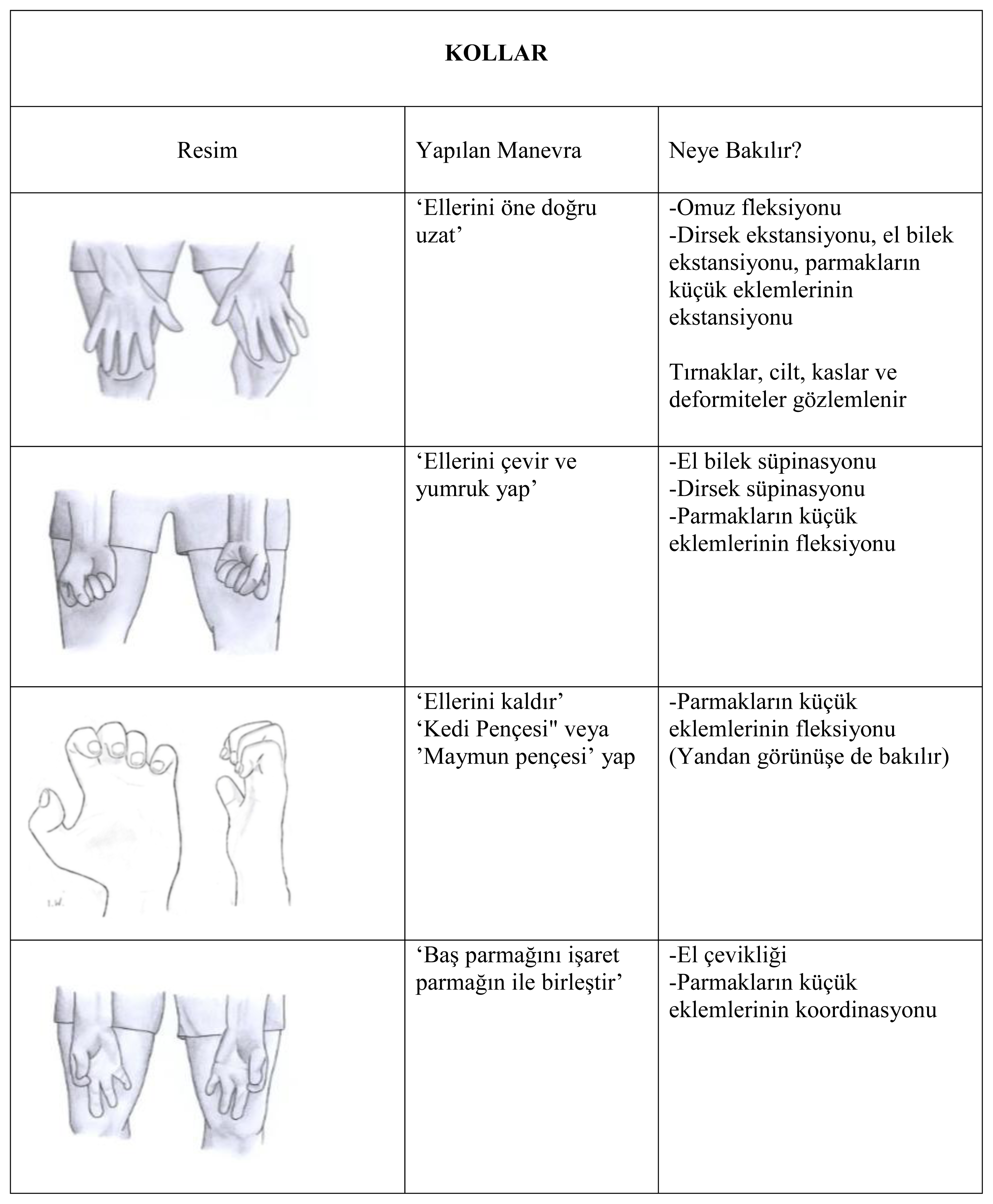

Supplement: Supplementary Figure — Turkish translation of video pGALS [file tjmed-55-01-348s1b.tif]

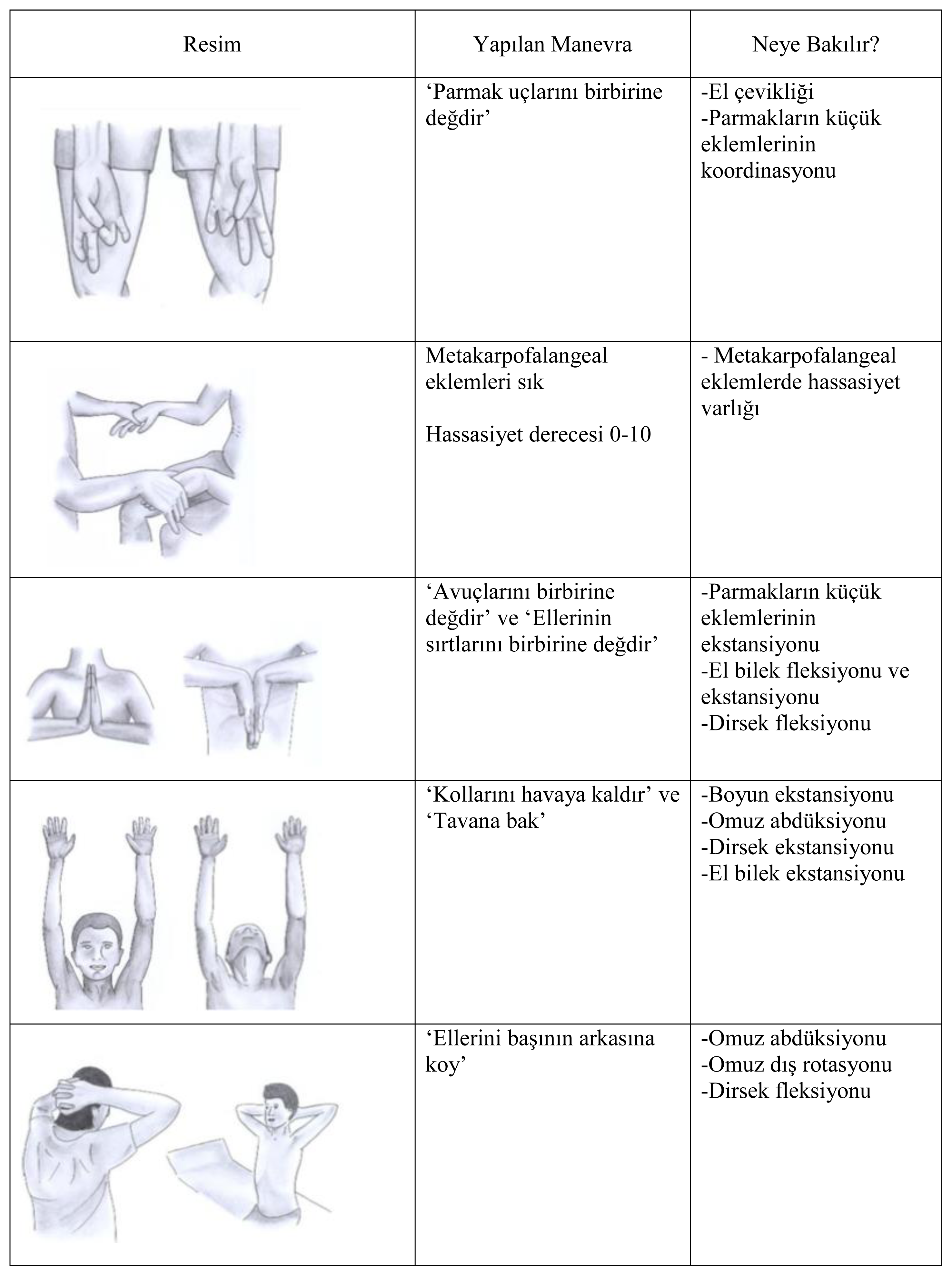

Supplement: Supplementary Figure — Turkish translation of video pGALS [file tjmed-55-01-348s1c.tif]

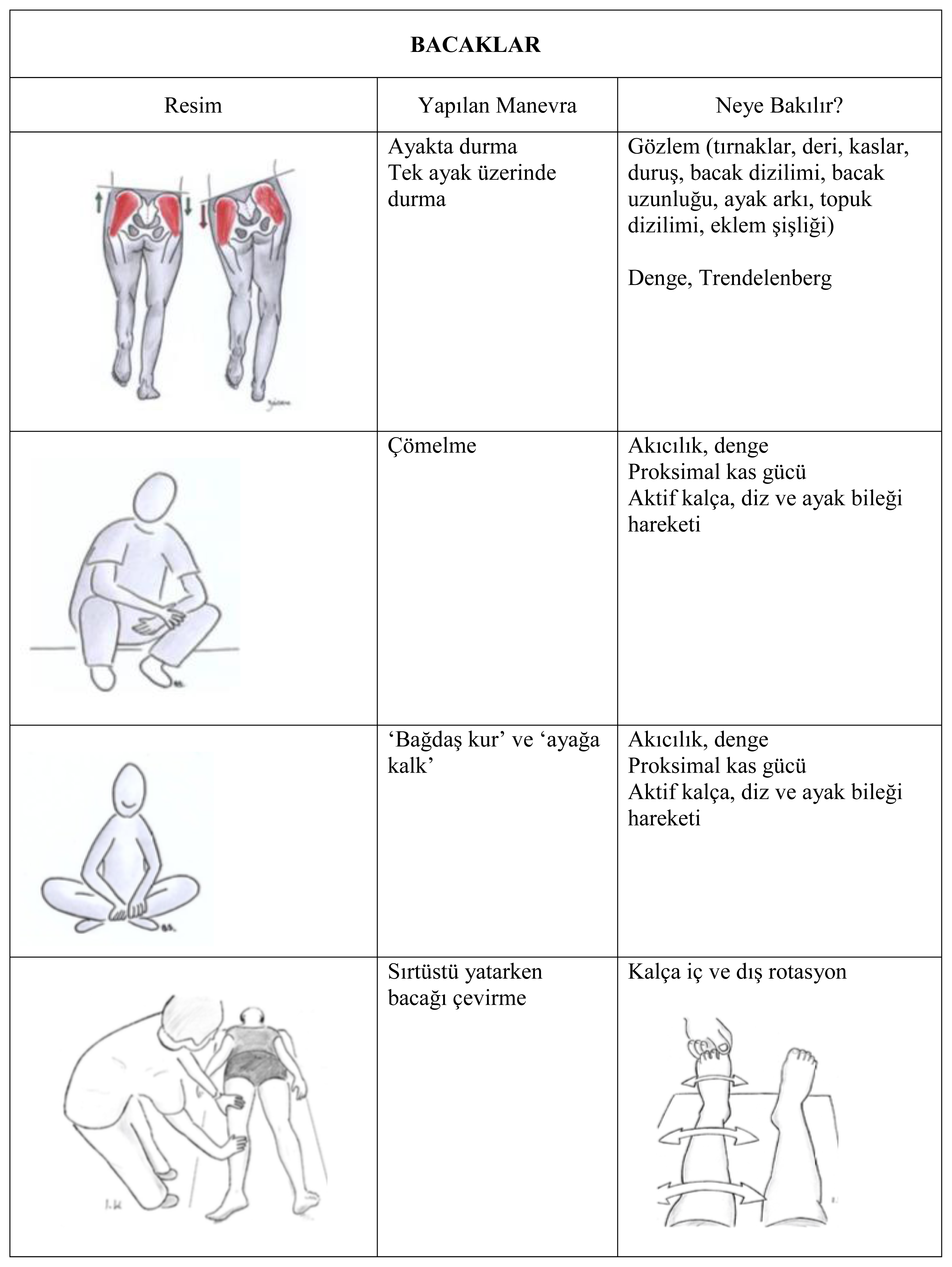

Supplement: Supplementary Figure — Turkish translation of video pGALS [file tjmed-55-01-348s1d.tif]

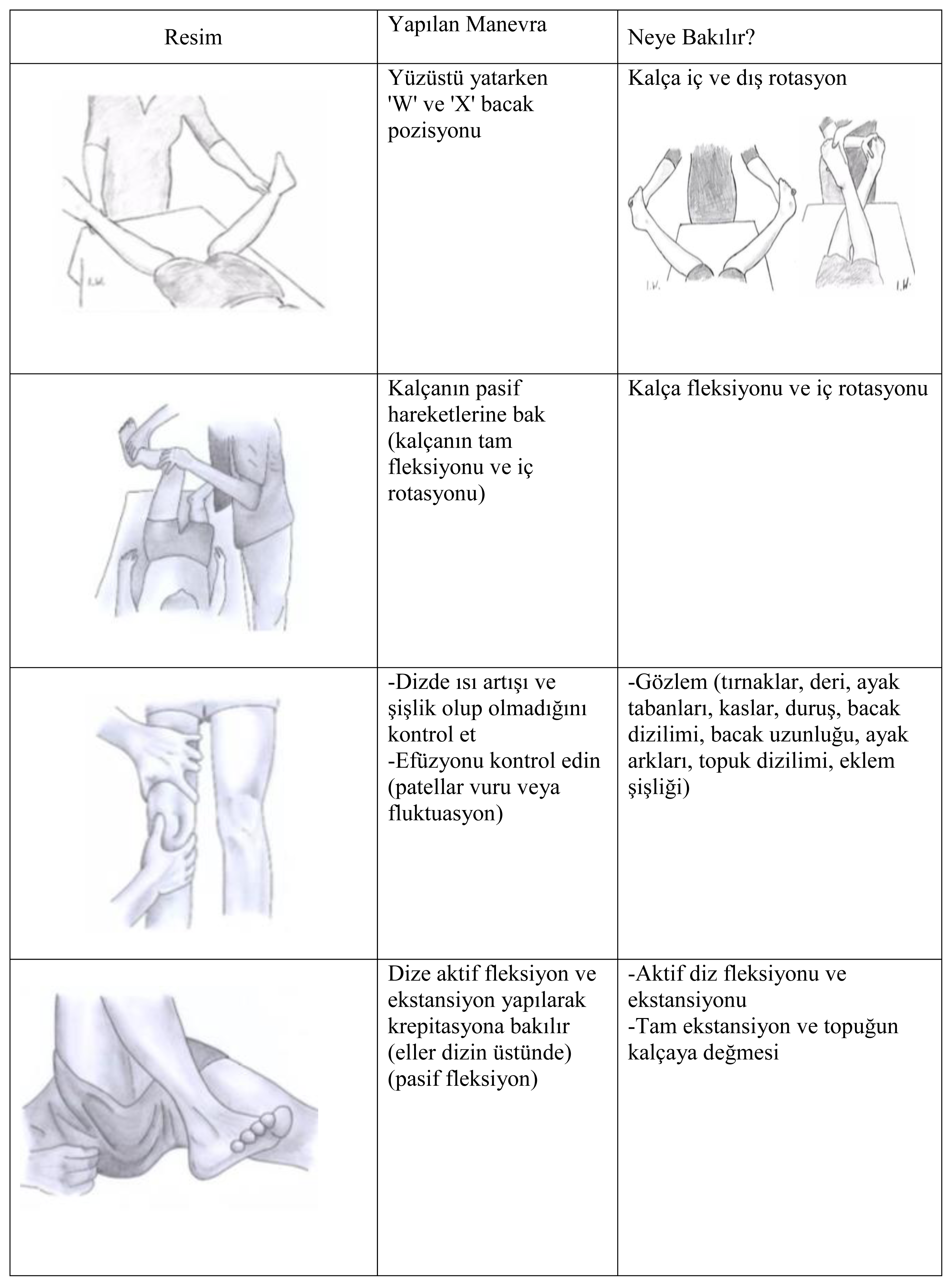

Supplement: Supplementary Figure — Turkish translation of video pGALS [file tjmed-55-01-348s1e.tif]

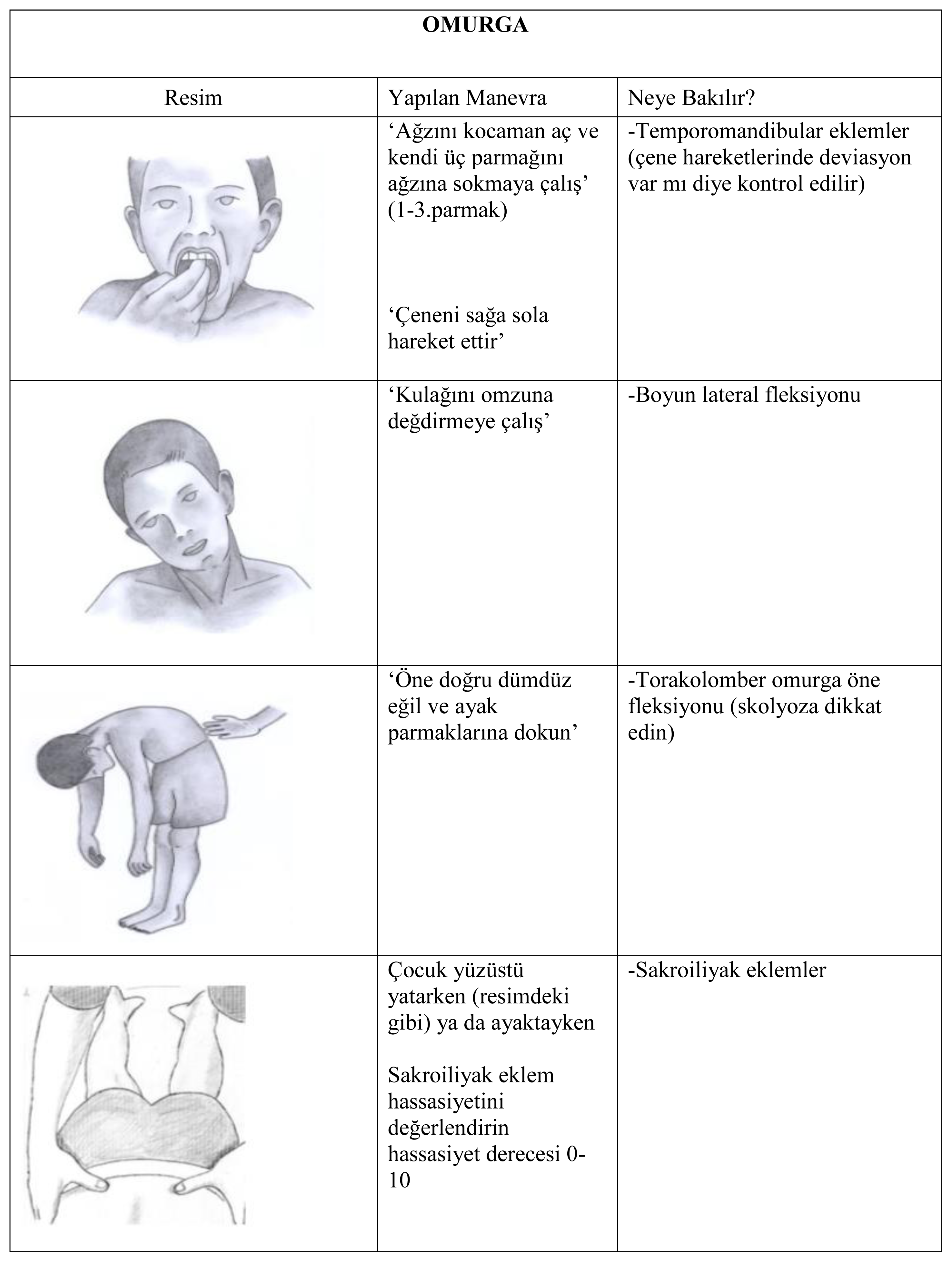

Supplement: Supplementary Figure — Turkish translation of video pGALS [file tjmed-55-01-348s1f.tif]
